# μ-Oxido-bis­[chlorido(4,4′-di-*tert*-butyl-2,2′-bipyridine-κ^2^ *N*,*N*′)dioxido­molybdenum(VI)] 0.2-hydrate

**DOI:** 10.1107/S1600536811046952

**Published:** 2011-11-12

**Authors:** Ana C. Gomes, José A. Fernandes, Carla A. Gamelas, Isabel S. Gonçalves, Filipe A. Almeida Paz

**Affiliations:** a Department of Chemistry, University of Aveiro, CICECO, 3810-193 Aveiro, Portugal; b Escola Superior de Tecnologia, Instituto Politécnico de Setúbal, 2910-761 Setúbal, Portugal

## Abstract

The title hydrate, [Mo_2_Cl_2_O_5_(C_18_H_24_N_2_)_2_]·0.2H_2_O, has been isolated as the oxidation product of [Mo(η^3^-C_3_H_5_)Cl(CO)_2_(di-*t*-Bu-bipy)] (where di-*t*-Bu-bipy is 4,4′-di-*tert*-butyl-2,2′-bipyridine). A μ-oxide ligand bridges two similar MoCl(di-*t*-Bu-bipy)O_2_ units, having the terminal oxide ligands mutually *cis*, and the chloride and μ-oxide *trans* to each other. In the binuclear complex, the coordination geometries of the metal atoms can be described as highly distorted octa­hedra. Individual complexes co-crystallize with a partially occupied water mol­ecule of crystallization (occupancy factor = 0.20; H atoms not located), with the crystal packing being mediated by the need to effectively fill the available space. A number of weak C—H⋯O and C—H⋯Cl inter­actions are present.

## Related literature

For general background to dioxidomolybdenum(VI) com­plexes, see: Arzoumanian *et al.* (2006 ▶); Jeyakumar & Chand (2009 ▶); Kühn *et al.* (2002 ▶); Rodrigues *et al.* (2004 ▶). For studies on molybdenum complexes from our research groups, see: Coelho *et al.* (2011 ▶); Fernandes *et al.* (2011*a* ▶,*b* ▶, 2011 ▶); Gago *et al.* (2009 ▶); Nunes *et al.* (2003 ▶); Pereira *et al.* (2007 ▶).
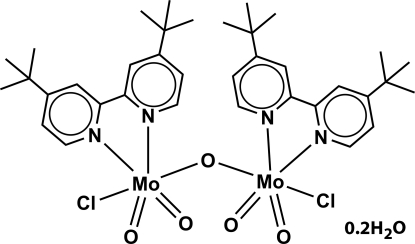


## Experimental

### 

#### Crystal data

[Mo_2_Cl_2_O_5_(C_18_H_24_N_2_)_2_]·0.2H_2_O
*M* *_r_* = 883.17
Monoclinic, 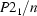

*a* = 16.9997 (7) Å
*b* = 12.7444 (6) Å
*c* = 18.4609 (8) Å
β = 99.582 (2)°
*V* = 3943.8 (3) Å^3^
*Z* = 4
Mo *K*α radiation
μ = 0.82 mm^−1^
*T* = 150 K
0.08 × 0.06 × 0.03 mm

#### Data collection

Bruker X8 KappaCCD APEXII diffractometer
Absorption correction: multi-scan (*SADABS*; Sheldrick, 1997 ▶) *T*_min_ = 0.938, *T*_max_ = 0.976
54239 measured reflections
10578 independent reflections
7469 reflections with *I* > 2σ(*I*)
*R*_int_ = 0.050

#### Refinement

*R*[*F* ^2^ > 2σ(*F* ^2^)] = 0.040
*wR*(*F* ^2^) = 0.086
*S* = 1.02
10578 reflections
463 parameters
18 restraints
H-atom parameters constrained
Δρ_max_ = 0.96 e Å^−3^
Δρ_min_ = −0.67 e Å^−3^

### 

Data collection: *APEX2* (Bruker, 2006 ▶); cell refinement: *SAINT-Plus* (Bruker, 2005 ▶); data reduction: *SAINT-Plus*; program(s) used to solve structure: *SHELXTL* (Sheldrick, 2008 ▶); program(s) used to refine structure: *SHELXTL*; molecular graphics: *DIAMOND* (Brandenburg, 2009 ▶); software used to prepare material for publication: *SHELXTL*.

## Supplementary Material

Crystal structure: contains datablock(s) global, I. DOI: 10.1107/S1600536811046952/tk5013sup1.cif

Structure factors: contains datablock(s) I. DOI: 10.1107/S1600536811046952/tk5013Isup2.hkl

Additional supplementary materials:  crystallographic information; 3D view; checkCIF report

## Figures and Tables

**Table 1:** Selected bond lengths (Å)

Mo1—O1	1.8920 (19)
Mo1—O2	1.6972 (19)
Mo1—O3	1.696 (2)
Mo1—N1	2.330 (2)
Mo1—N2	2.323 (2)
Mo1—Cl1	2.4895 (8)
Mo2—O1	1.9274 (19)
Mo2—O4	1.6975 (19)
Mo2—O5	1.694 (2)
Mo2—N3	2.328 (2)
Mo2—N4	2.304 (2)
Mo2—Cl2	2.4283 (8)

**Table 2:** Hydrogen-bond geometry (Å, °)

*D*—H⋯*A*	*D*—H	H⋯*A*	*D*⋯*A*	*D*—H⋯*A*
C27—H27⋯O1^i^	0.95	2.52	3.341 (3)	145
C34—H34*A*⋯Cl1^ii^	0.98	2.77	3.748 (4)	174
C35—H35*A*⋯O4^i^	0.98	2.54	3.421 (4)	149
C12—H12*C*⋯O1*W*	0.98	2.69	3.641 (16)	163
C18—H18*B*⋯O1*W*	0.98	2.10	2.970 (18)	147
O1*W*⋯Cl2^i^			3.573 (18)	
O1*W*⋯O5^iii^			3.236 (17)	

Symmetry codes: (i) 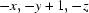; (ii) 
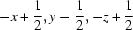
; (iii) 
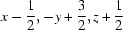
.

**Fig. 1.**
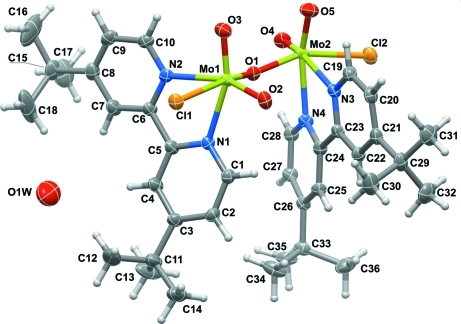
Asymmetric unit of the title compound showing all non-hydrogen atoms represented as thermal ellipsoids drawn at the 50% probability level. The water molecule has a site occupancy factor = 0.20. Hydrogen atoms are represented as small spheres with arbitrary radii and the atomic labeling is provided for all non-hydrogen atoms.

**Fig. 2.**
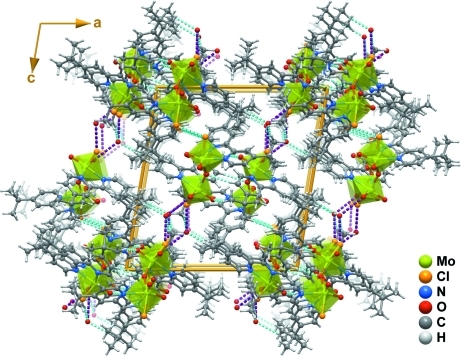
Crystal packing of the title compound viewed in perspective along [010] direction. The highly distorted {MoCl2N2O2} coordination polyhedra are represented as translucent octahedra for clarity. Supramolecular contacts interconnecting adjacent chemical moieties are represented as dashed lines: C—H···O and C—H···Cl in light blue; Owater···O and Owater···Cl in violet.

## supplementary crystallographic information

### Comment

Dioxomolybdenum(VI) complexes are known to be highly active catalysts in the epoxidation of olefins (Kühn *et al.*, 2002; Jeyakumar & Chand, 2009). In these complexes the active metal-oxo functional group may appear with two distinct structural motifs: as a terminal oxo (Mo═O) or as a bridging µ-oxo (Mo—O—Mo). Compounds with the latter type of bridging group are significantly less studied but have been shown to be intermediates in a handful of interesting catalytic systems (Nunes *et al.*, 2003). Following our on-going interest in the study of this type of family of compounds (Fernandes *et al.*, 2010*a*,*b*, 2011) we have recently described the synthesis and structural details of the oxo-µ-oxo complexes [Mo_2_O_4_(µ-O)Cl_2_(DMF)_4_] (Gago *et al.*, 2009), [Mo_2_O_4_(µ-O)Cl_2_(pyrazole)_4_] (Pereira *et al.*, 2007), and [Mo_2_O_4_(µ-O)Cl_2_(PzPy)_2_] (where PzPy stands for 2-(3-pyrazolyl)pyridine) (Coelho *et al.*, 2011). Noteworthy, these complexes were found to be highly active in epoxidation catalysis with *tert*-butylhydroperoxide. The title compound, a µ-oxo dimer with empirical formula [Mo_2_O_4_(µ-O)Cl_2_(di-*t*-Bu-bipy)_2_] (where di-*t*-Bu-bipy stands for 4,4'-di-*tert*-butyl-2,2'-bipyridine) which simultaneously contains terminal Mo═O oxo groups and a bridging µ-oxo one, has been recently synthesized by Arzoumanian *et al.* (2006) and we now wish to report its crystal structure at the low temperature of 150 K.

The asymmetric unit of the title compound comprises a whole binuclear molecular entity, C_36_H_48_Cl_2_Mo_2_N_4_O_5_, and a partially occupied (20%) water molecule of crystallization. The binuclear complex is formed by two crystallographically independent Mo(VI) centres bridged *via* a µ-oxo group imposing a Mo···Mo distance of 3.6273 (4) Å. The chemical environment of these metallic centers is very similar, being composed of a pair of *cis*-positioned terminal oxo ligands, a chlorido and a *N,N*-chelating 4,4'-di-*tert*-butyl-2,2'-bipyridine (di-*t*-Bu-bipy) molecule as depicted in Fig. 1. The coordination environments around the metal centers can be described as highly distorted octahedra due to, on the one hand, the existence of chlorido ligands (*trans*-positioned with respect to the µ-oxo ligand) and, on the other, to the typical *trans* effect of the Mo═O groups: while the Mo—O_bridge_ distances are 1.8920 (19) and 1.9274 (19) Å, the Mo—O_terminal_ distances range from 1.694 (2) to 1.6975 (19) Å; the Mo—Cl distances are 2.4895 (8) and 2.4283 (8) Å and the Mo—N bonds range from 2.304 (2) to 2.330 (2) Å. The *cis* and *trans* octahedral angles are in the ranges of 68.95 (8) to 107.35 (10)° and 157.51 (6) to 160.69 (9)°, respectively. The Mo1—O1—Mo2 kink angle through the µ-oxo bridge is 143.50 (10)° which, to the best of our knowledge, constitutes the smallest reported to date for related binuclear dioxomolybdenum(VI) complexes: the analogous value for [Mo_2_O_4_(µ-O)Cl_2_(DMF)_4_] is *ca* 175° and that for [Mo_2_O_4_(µ-O)Cl_2_(pyrazole)_4_] is *ca* 151°, and those for the two conformers of [Mo_2_O_4_(µ-O)Cl_2_(PzPy)_2_] are *ca* 156 and 180°. We attribute this structural feature to the considerable steric hindrance associated with the di-*t*-Bu-bipy moieties, mostly due to the pendant —CH_3_ groups. In this context, the two average planes containing the aromatic rings of the two crystallographically independent di-*t*-Bu-bipy molecules subtend an angle of *ca* 34°, which contrasts with the parallel nature observed for the two conformers of [Mo_2_O_4_(µ-O)Cl_2_(PzPy)_2_]. Noteworthy, the torsion angles N1—Mo1···Mo2—N4 and N2—Mo1···Mo2—N3 are -18.40 (7) and -157.03 (9)°, respectively.

The crystal packing is mainly driven by the need to effectively fill the available space (van de Waals contacts) in conjunction with several weak supramolecular interactions, namely weak C—H···O and C—H···Cl hydrogen bonding interactions (light blue dashed lines in Fig. 2; see Table 2 for geometric details). The water molecule of crystallization (O1W), which is only statistically present in 1/5 of the asymmetric units, accepts the hydrogen donation from adjacent C—H groups and also acts as hydrogen bond donor to Cl2 and O5 of neighboring molecules (violet dashed lines in Figure 2; see Table 2 for geometrical details). Even though the location of the water molecule permits its full site occupancy, we postulate that the absence of suitable hydrogen bonding partners in the binuclear complexes contributes significantly for its partial occupancy in the crystal structure.

### Experimental

Chemicals were purchased from commercial sources and used as received. The compound [Mo(η^3^-C_3_H_5_)Cl(CO)_2_(di-*t*-Bu-bipy)] (1) was prepared following a literature method (Rodrigues *et al.*, 2004). Thus, 70% aqueous *tert*-butylhydroperoxide (TBHP) (0.64 mL, 4.60 mmol) was added dropwise to a stirred solution of 1 (0.23 g, 0.46 mmol) in CH_3_CN (20 mL). After stirring at ambient temperature for 15 h, the resultant yellow solution was filtered off, concentrated, and a very pale yellow solid precipitated after the addition of *n*-hexane and diethyl ether. The precipitate was filtered, washed with *n*-hexane and diethyl ether, and vacuum-dried. Yield: 0.14 g, 69%.

The same product (as confirmed by a comparison of FT–IR and ^1^H NMR spectra, and microanalysis data) was obtained by using a decane solution of TBHP (5–6 M, 10 equiv.) instead of the aqueous solution, with 1 dissolved in CH_2_Cl_2_ under otherwise similar conditions (the excess of TBHP was destroyed with MnO_2_).

Anal. Calcd. for C_36_H_48_N_4_Cl_2_Mo_2_O_5_.0.2H_2_O (in %): C, 48.96; H, 5.52; N, 6.34. Found (in %): C, 49.47; H, 5.52; N, 6.28. The FT–IR and ^1^H NMR spectral data were in agreement with published data (Arzoumanian *et al.*, 2006).

Suitable crystals were obtained by the slow diffusion of diethyl ether into a concentrated solution of the compound in CH_2_Cl_2_ with a small layer of *n*-hexane.

### Refinement

Hydrogen atoms bound to carbon were placed in idealized positions and were included in the final structural model in riding-motion approximation with C—H = 0.95 Å (aromatic C—H) and 0.98 Å (—CH_3_). The isotropic thermal displacement parameters for these atoms were fixed at 1.2×*U*_eq_ (aromatic C—H) or 1.5×*U*_eq_ (—CH_3_) of the respective parent carbon atoms.

One water molecule of crystallization was found to be partially occupied and was included in the final structural model with fixed rate of occupancy of 20% (calculated from unrestrained refinement for the site occupancy). Hydrogen atoms associated with this water molecule could not be located from difference Fourier maps and attempts to include these in calculated positions did not lead stable structural refinements. Nevertheless, the hydrogen atoms associated with this chemical entity have been included in the empirical formula of the title compound.

### Crystal data

**Table:**

[Mo_2_Cl_2_O_5_(C_18_H_24_N_2_)_2_]·0.2H_2_O	*F*(000) = 1808
*M**_r_* = 883.17	*D*_x_ = 1.487 Mg m^−^^3^
Monoclinic, *P*2_1_/*n*	Mo *K*α radiation, λ = 0.71073 Å
Hall symbol: -P 2yn	Cell parameters from 9903 reflections
*a* = 16.9997 (7) Å	θ = 2.8–29.1°
*b* = 12.7444 (6) Å	µ = 0.82 mm^−^^1^
*c* = 18.4609 (8) Å	*T* = 150 K
β = 99.582 (2)°	Block, yellow
*V* = 3943.8 (3) Å^3^	0.08 × 0.06 × 0.03 mm
*Z* = 4	

### Data collection

**Table:**

Bruker X8 KappaCCD APEXII diffractometer	10578 independent reflections
Radiation source: fine-focus sealed tube	7469 reflections with *I* > 2σ(*I*)
graphite	*R*_int_ = 0.050
ω and φ scans	θ_max_ = 29.1°, θ_min_ = 3.5°
Absorption correction: multi-scan (*SADABS*; Sheldrick, 1997)	*h* = −23→23
*T*_min_ = 0.938, *T*_max_ = 0.976	*k* = −16→17
54239 measured reflections	*l* = −24→25

### Refinement

**Table:**

Refinement on *F*^2^	Primary atom site location: structure-invariant direct methods
Least-squares matrix: full	Secondary atom site location: difference Fourier map
*R*[*F*^2^ > 2σ(*F*^2^)] = 0.040	Hydrogen site location: inferred from neighbouring sites
*wR*(*F*^2^) = 0.086	H-atom parameters constrained
*S* = 1.02	*w* = 1/[σ^2^(*F*_o_^2^) + (0.0315*P*)^2^ + 3.406*P*] where *P* = (*F*_o_^2^ + 2*F*_c_^2^)/3
10578 reflections	(Δ/σ)_max_ = 0.001
463 parameters	Δρ_max_ = 0.96 e Å^−^^3^
18 restraints	Δρ_min_ = −0.67 e Å^−^^3^

### Special details

**Table:**

Geometry. All e.s.d.'s (except the e.s.d. in the dihedral angle between two l.s. planes) are estimated using the full covariance matrix. The cell e.s.d.'s are taken into account individually in the estimation of e.s.d.'s in distances, angles and torsion angles; correlations between e.s.d.'s in cell parameters are only used when they are defined by crystal symmetry. An approximate (isotropic) treatment of cell e.s.d.'s is used for estimating e.s.d.'s involving l.s. planes.
Refinement. Refinement of *F*^2^ against ALL reflections. The weighted *R*-factor *wR* and goodness of fit *S* are based on *F*^2^, conventional *R*-factors *R* are based on *F*, with *F* set to zero for negative *F*^2^. The threshold expression of *F*^2^ > σ(*F*^2^) is used only for calculating *R*-factors(gt) *etc*. and is not relevant to the choice of reflections for refinement. *R*-factors based on *F*^2^ are statistically about twice as large as those based on *F*, and *R*- factors based on ALL data will be even larger.

### Fractional atomic coordinates and isotropic or equivalent isotropic displacement parameters (Å^2^)

**Table:**

	*x*	*y*	*z*	*U*_iso_*/*U*_eq_	Occ. (<1)
Mo1	0.161140 (13)	0.78289 (2)	0.101763 (13)	0.02009 (7)	
Mo2	0.153567 (14)	0.63578 (2)	−0.067460 (14)	0.02215 (7)	
Cl1	0.16494 (4)	0.87386 (6)	0.22163 (4)	0.02652 (16)	
Cl2	0.21012 (5)	0.51282 (7)	−0.14517 (4)	0.03473 (19)	
N1	0.11178 (12)	0.65491 (18)	0.17242 (13)	0.0210 (5)	
N2	0.02749 (13)	0.81233 (19)	0.10764 (13)	0.0213 (5)	
N3	0.27507 (13)	0.59963 (19)	0.00696 (13)	0.0223 (5)	
N4	0.14638 (12)	0.48235 (19)	−0.00290 (12)	0.0211 (5)	
O1	0.12571 (10)	0.69242 (15)	0.02171 (10)	0.0224 (4)	
O2	0.25605 (11)	0.73982 (17)	0.12818 (11)	0.0276 (5)	
O3	0.16755 (12)	0.89784 (16)	0.05679 (12)	0.0311 (5)	
O4	0.06091 (11)	0.61851 (17)	−0.11680 (11)	0.0297 (5)	
O5	0.19330 (13)	0.74344 (18)	−0.10133 (12)	0.0360 (5)	
C1	0.15948 (16)	0.5835 (2)	0.21063 (16)	0.0238 (6)	
H1	0.2115	0.5743	0.1992	0.029*	
C2	0.13739 (16)	0.5228 (2)	0.26543 (17)	0.0245 (6)	
H2	0.1739	0.4736	0.2910	0.029*	
C3	0.06192 (16)	0.5334 (2)	0.28335 (16)	0.0263 (7)	
C4	0.01067 (16)	0.6030 (3)	0.23986 (17)	0.0297 (7)	
H4	−0.0432	0.6088	0.2472	0.036*	
C5	0.03666 (15)	0.6629 (2)	0.18700 (16)	0.0235 (6)	
C6	−0.01214 (16)	0.7471 (2)	0.14631 (16)	0.0232 (6)	
C7	−0.09241 (16)	0.7619 (3)	0.14974 (17)	0.0292 (7)	
H7	−0.1198	0.7126	0.1751	0.035*	
C8	−0.13296 (16)	0.8487 (3)	0.11624 (17)	0.0298 (7)	
C9	−0.09027 (17)	0.9162 (3)	0.07927 (17)	0.0295 (7)	
H9	−0.1152	0.9770	0.0561	0.035*	
C10	−0.01101 (17)	0.8954 (2)	0.07589 (16)	0.0245 (6)	
H10	0.0171	0.9427	0.0497	0.029*	
C11	0.03393 (19)	0.4783 (3)	0.34808 (19)	0.0385 (8)	
C12	0.0029 (3)	0.5616 (4)	0.3962 (2)	0.0777 (16)	
H12A	0.0464	0.6095	0.4158	0.117*	
H12B	−0.0172	0.5273	0.4370	0.117*	
H12C	−0.0403	0.6013	0.3666	0.117*	
C13	−0.0336 (2)	0.4023 (4)	0.3180 (2)	0.0666 (14)	
H13A	−0.0763	0.4408	0.2868	0.100*	
H13B	−0.0547	0.3701	0.3590	0.100*	
H13C	−0.0129	0.3474	0.2890	0.100*	
C14	0.1016 (2)	0.4186 (3)	0.3952 (2)	0.0461 (10)	
H14A	0.1204	0.3627	0.3659	0.069*	
H14B	0.0822	0.3878	0.4376	0.069*	
H14C	0.1456	0.4669	0.4123	0.069*	
C15	−0.22161 (17)	0.8645 (3)	0.1206 (2)	0.0397 (9)	
C16	−0.2495 (2)	0.9713 (4)	0.0924 (4)	0.0971 (19)	
H16A	−0.3076	0.9757	0.0886	0.146*	
H16B	−0.2348	0.9824	0.0438	0.146*	
H16C	−0.2243	1.0252	0.1264	0.146*	
C17	−0.2693 (2)	0.7825 (4)	0.0698 (3)	0.0681 (13)	
H17A	−0.2527	0.7118	0.0870	0.102*	
H17B	−0.2590	0.7920	0.0195	0.102*	
H17C	−0.3264	0.7914	0.0706	0.102*	
C18	−0.2369 (3)	0.8407 (6)	0.1960 (3)	0.106 (2)	
H18A	−0.2121	0.8948	0.2301	0.159*	
H18B	−0.2142	0.7720	0.2115	0.159*	
H18C	−0.2946	0.8397	0.1960	0.159*	
C19	0.33976 (16)	0.6593 (2)	0.00679 (17)	0.0274 (7)	
H19	0.3352	0.7203	−0.0232	0.033*	
C20	0.41289 (16)	0.6358 (3)	0.04836 (17)	0.0279 (7)	
H20	0.4572	0.6806	0.0465	0.033*	
C21	0.42233 (15)	0.5476 (2)	0.09269 (16)	0.0241 (6)	
C22	0.35406 (15)	0.4888 (2)	0.09491 (16)	0.0243 (6)	
H22	0.3568	0.4295	0.1265	0.029*	
C23	0.28184 (15)	0.5150 (2)	0.05182 (15)	0.0206 (6)	
C24	0.20800 (15)	0.4519 (2)	0.04866 (15)	0.0209 (6)	
C25	0.20205 (15)	0.3673 (2)	0.09379 (15)	0.0213 (6)	
H25	0.2459	0.3494	0.1307	0.026*	
C26	0.13233 (16)	0.3073 (2)	0.08606 (15)	0.0211 (6)	
C27	0.07065 (16)	0.3385 (2)	0.03097 (16)	0.0234 (6)	
H27	0.0223	0.2995	0.0223	0.028*	
C28	0.07950 (15)	0.4252 (2)	−0.01084 (16)	0.0232 (6)	
H28	0.0360	0.4457	−0.0473	0.028*	
C29	0.50289 (16)	0.5150 (3)	0.13790 (18)	0.0304 (7)	
C30	0.50366 (18)	0.5490 (3)	0.21771 (19)	0.0435 (9)	
H30A	0.5534	0.5256	0.2481	0.065*	
H30B	0.4582	0.5174	0.2359	0.065*	
H30C	0.4999	0.6256	0.2201	0.065*	
C31	0.57226 (17)	0.5677 (3)	0.1089 (2)	0.0420 (9)	
H31A	0.5688	0.6439	0.1147	0.063*	
H31B	0.5696	0.5505	0.0568	0.063*	
H31C	0.6229	0.5423	0.1367	0.063*	
C32	0.51307 (18)	0.3958 (3)	0.1352 (2)	0.0428 (9)	
H32A	0.5029	0.3722	0.0840	0.064*	
H32B	0.4752	0.3619	0.1624	0.064*	
H32C	0.5676	0.3770	0.1575	0.064*	
C33	0.12703 (17)	0.2135 (2)	0.13614 (16)	0.0260 (6)	
C34	0.1347 (2)	0.2522 (3)	0.21501 (17)	0.0394 (9)	
H34A	0.1857	0.2888	0.2288	0.059*	
H34B	0.1325	0.1923	0.2479	0.059*	
H34C	0.0908	0.3006	0.2192	0.059*	
C35	0.04774 (18)	0.1555 (3)	0.11683 (17)	0.0319 (7)	
H35A	0.0043	0.2012	0.1265	0.048*	
H35B	0.0490	0.0919	0.1469	0.048*	
H35C	0.0391	0.1362	0.0647	0.048*	
C36	0.1950 (2)	0.1374 (3)	0.1283 (2)	0.0459 (9)	
H36A	0.1899	0.1143	0.0771	0.069*	
H36B	0.1922	0.0763	0.1601	0.069*	
H36C	0.2464	0.1729	0.1428	0.069*	
O1W	−0.1847 (9)	0.6639 (13)	0.2975 (10)	0.078 (5)	0.20

### Atomic displacement parameters (Å^2^)

**Table:**

	*U*^11^	*U*^22^	*U*^33^	*U*^12^	*U*^13^	*U*^23^
Mo1	0.01580 (10)	0.02716 (14)	0.01656 (13)	−0.00222 (10)	0.00054 (8)	−0.00064 (11)
Mo2	0.02064 (11)	0.02864 (15)	0.01613 (13)	0.00094 (10)	0.00001 (9)	0.00249 (11)
Cl1	0.0231 (3)	0.0348 (4)	0.0201 (4)	−0.0005 (3)	−0.0010 (3)	−0.0053 (3)
Cl2	0.0354 (4)	0.0461 (5)	0.0232 (4)	0.0068 (4)	0.0064 (3)	−0.0019 (4)
N1	0.0157 (10)	0.0261 (14)	0.0201 (13)	−0.0015 (9)	0.0001 (9)	−0.0008 (10)
N2	0.0193 (11)	0.0258 (14)	0.0169 (13)	0.0015 (9)	−0.0026 (9)	−0.0009 (10)
N3	0.0197 (11)	0.0278 (14)	0.0197 (13)	−0.0008 (10)	0.0042 (9)	−0.0008 (10)
N4	0.0175 (10)	0.0289 (14)	0.0150 (12)	−0.0024 (9)	−0.0030 (9)	0.0006 (10)
O1	0.0183 (9)	0.0286 (12)	0.0188 (11)	0.0011 (8)	−0.0015 (8)	−0.0022 (8)
O2	0.0177 (9)	0.0421 (13)	0.0222 (11)	−0.0017 (9)	0.0009 (8)	−0.0067 (9)
O3	0.0377 (12)	0.0301 (12)	0.0259 (12)	−0.0038 (9)	0.0067 (10)	0.0005 (9)
O4	0.0260 (10)	0.0400 (13)	0.0199 (11)	0.0023 (9)	−0.0052 (8)	−0.0001 (9)
O5	0.0374 (12)	0.0373 (14)	0.0332 (13)	−0.0015 (10)	0.0059 (10)	0.0100 (11)
C1	0.0184 (12)	0.0276 (17)	0.0244 (16)	0.0005 (11)	0.0008 (11)	−0.0013 (13)
C2	0.0202 (13)	0.0260 (16)	0.0257 (17)	0.0038 (11)	−0.0011 (12)	0.0026 (13)
C3	0.0223 (13)	0.0332 (18)	0.0221 (16)	0.0011 (12)	0.0001 (12)	0.0064 (13)
C4	0.0164 (13)	0.043 (2)	0.0296 (18)	0.0021 (12)	0.0049 (12)	0.0090 (14)
C5	0.0167 (12)	0.0327 (17)	0.0204 (15)	−0.0003 (11)	0.0008 (11)	0.0024 (13)
C6	0.0195 (13)	0.0300 (17)	0.0189 (15)	0.0008 (11)	−0.0007 (11)	0.0022 (12)
C7	0.0168 (13)	0.044 (2)	0.0259 (17)	0.0010 (13)	0.0016 (12)	0.0048 (14)
C8	0.0201 (13)	0.043 (2)	0.0248 (17)	0.0051 (13)	−0.0012 (12)	−0.0036 (14)
C9	0.0282 (15)	0.0311 (18)	0.0256 (17)	0.0071 (13)	−0.0059 (13)	0.0003 (14)
C10	0.0277 (14)	0.0245 (16)	0.0189 (16)	−0.0003 (12)	−0.0032 (12)	−0.0014 (12)
C11	0.0338 (17)	0.051 (2)	0.033 (2)	0.0093 (16)	0.0107 (14)	0.0173 (17)
C12	0.107 (4)	0.090 (4)	0.047 (3)	0.053 (3)	0.043 (3)	0.033 (3)
C13	0.0322 (19)	0.099 (4)	0.070 (3)	−0.011 (2)	0.0104 (19)	0.048 (3)
C14	0.0408 (19)	0.058 (3)	0.040 (2)	0.0081 (17)	0.0077 (16)	0.0235 (19)
C15	0.0185 (14)	0.059 (2)	0.040 (2)	0.0117 (15)	0.0008 (13)	0.0013 (18)
C16	0.037 (2)	0.072 (3)	0.186 (6)	0.021 (2)	0.029 (3)	0.006 (4)
C17	0.0261 (17)	0.096 (4)	0.083 (3)	−0.005 (2)	0.0089 (19)	−0.006 (3)
C18	0.048 (2)	0.219 (6)	0.056 (3)	0.055 (3)	0.023 (2)	0.010 (4)
C19	0.0246 (14)	0.0271 (17)	0.0318 (18)	0.0008 (12)	0.0084 (13)	0.0010 (14)
C20	0.0179 (13)	0.0360 (18)	0.0302 (18)	−0.0029 (12)	0.0057 (12)	−0.0050 (14)
C21	0.0188 (13)	0.0330 (18)	0.0198 (16)	0.0008 (12)	0.0013 (11)	−0.0062 (13)
C22	0.0201 (13)	0.0294 (17)	0.0230 (16)	0.0016 (12)	0.0025 (11)	−0.0003 (13)
C23	0.0175 (12)	0.0281 (16)	0.0160 (15)	0.0000 (11)	0.0020 (10)	−0.0008 (12)
C24	0.0186 (12)	0.0260 (16)	0.0163 (15)	−0.0006 (11)	−0.0024 (10)	−0.0034 (12)
C25	0.0201 (12)	0.0250 (16)	0.0166 (14)	−0.0008 (11)	−0.0033 (11)	0.0003 (12)
C26	0.0221 (13)	0.0240 (16)	0.0161 (15)	−0.0014 (11)	0.0000 (11)	−0.0022 (12)
C27	0.0204 (13)	0.0268 (17)	0.0212 (16)	−0.0040 (11)	−0.0018 (11)	−0.0022 (12)
C28	0.0189 (12)	0.0306 (17)	0.0178 (15)	−0.0008 (11)	−0.0039 (11)	−0.0023 (13)
C29	0.0168 (13)	0.045 (2)	0.0282 (18)	0.0030 (13)	0.0011 (12)	−0.0047 (15)
C30	0.0240 (15)	0.072 (3)	0.031 (2)	0.0066 (16)	−0.0046 (14)	−0.0089 (18)
C31	0.0182 (14)	0.064 (3)	0.043 (2)	0.0004 (15)	0.0031 (14)	0.0020 (19)
C32	0.0232 (15)	0.052 (2)	0.051 (2)	0.0094 (15)	−0.0012 (15)	−0.0005 (18)
C33	0.0273 (14)	0.0306 (17)	0.0182 (15)	−0.0062 (13)	−0.0013 (11)	0.0023 (13)
C34	0.0481 (19)	0.046 (2)	0.0210 (18)	−0.0263 (16)	−0.0025 (15)	−0.0002 (15)
C35	0.0388 (17)	0.0327 (19)	0.0199 (17)	−0.0130 (14)	−0.0075 (13)	0.0037 (14)
C36	0.0426 (19)	0.034 (2)	0.060 (3)	0.0063 (16)	0.0040 (18)	0.0164 (18)
O1W	0.055 (9)	0.079 (12)	0.105 (14)	−0.006 (8)	0.029 (9)	0.015 (10)

### Geometric parameters (Å, °)

**Table:**

Mo1—O1	1.8920 (19)	C15—C17	1.541 (5)
Mo1—O2	1.6972 (19)	C16—H16A	0.9800
Mo1—O3	1.696 (2)	C16—H16B	0.9800
Mo1—N1	2.330 (2)	C16—H16C	0.9800
Mo1—N2	2.323 (2)	C17—H17A	0.9800
Mo1—Cl1	2.4895 (8)	C17—H17B	0.9800
Mo2—O1	1.9274 (19)	C17—H17C	0.9800
Mo2—O4	1.6975 (19)	C18—H18A	0.9800
Mo2—O5	1.694 (2)	C18—H18B	0.9800
Mo2—N3	2.328 (2)	C18—H18C	0.9800
Mo2—N4	2.304 (2)	C19—C20	1.381 (4)
Mo2—Cl2	2.4283 (8)	C19—H19	0.9500
N1—C1	1.339 (3)	C20—C21	1.384 (4)
N1—C5	1.352 (3)	C20—H20	0.9500
N2—C10	1.329 (4)	C21—C22	1.388 (4)
N2—C6	1.347 (4)	C21—C29	1.537 (4)
N3—C19	1.337 (4)	C22—C23	1.388 (4)
N3—C23	1.353 (4)	C22—H22	0.9500
N4—C28	1.338 (3)	C23—C24	1.484 (4)
N4—C24	1.350 (3)	C24—C25	1.377 (4)
C1—C2	1.375 (4)	C25—C26	1.397 (4)
C1—H1	0.9500	C25—H25	0.9500
C2—C3	1.384 (4)	C26—C27	1.391 (4)
C2—H2	0.9500	C26—C33	1.523 (4)
C3—C4	1.399 (4)	C27—C28	1.371 (4)
C3—C11	1.529 (4)	C27—H27	0.9500
C4—C5	1.369 (4)	C28—H28	0.9500
C4—H4	0.9500	C29—C31	1.529 (4)
C5—C6	1.482 (4)	C29—C32	1.531 (5)
C6—C7	1.389 (4)	C29—C30	1.534 (5)
C7—C8	1.393 (4)	C30—H30A	0.9800
C7—H7	0.9500	C30—H30B	0.9800
C8—C9	1.378 (4)	C30—H30C	0.9800
C8—C15	1.536 (4)	C31—H31A	0.9800
C9—C10	1.385 (4)	C31—H31B	0.9800
C9—H9	0.9500	C31—H31C	0.9800
C10—H10	0.9500	C32—H32A	0.9800
C11—C14	1.524 (4)	C32—H32B	0.9800
C11—C13	1.533 (5)	C32—H32C	0.9800
C11—C12	1.534 (6)	C33—C34	1.522 (4)
C12—H12A	0.9800	C33—C35	1.526 (4)
C12—H12B	0.9800	C33—C36	1.534 (4)
C12—H12C	0.9800	C34—H34A	0.9800
C13—H13A	0.9800	C34—H34B	0.9800
C13—H13B	0.9800	C34—H34C	0.9800
C13—H13C	0.9800	C35—H35A	0.9800
C14—H14A	0.9800	C35—H35B	0.9800
C14—H14B	0.9800	C35—H35C	0.9800
C14—H14C	0.9800	C36—H36A	0.9800
C15—C18	1.490 (6)	C36—H36B	0.9800
C15—C16	1.504 (6)	C36—H36C	0.9800
			
O3—Mo1—O2	106.53 (10)	C18—C15—C17	105.9 (4)
O3—Mo1—O1	100.49 (9)	C16—C15—C17	107.4 (4)
O2—Mo1—O1	101.02 (9)	C8—C15—C17	107.5 (3)
O3—Mo1—N2	91.51 (9)	C15—C16—H16A	109.5
O2—Mo1—N2	158.31 (9)	C15—C16—H16B	109.5
O1—Mo1—N2	87.03 (8)	H16A—C16—H16B	109.5
O3—Mo1—N1	159.54 (9)	C15—C16—H16C	109.5
O2—Mo1—N1	91.52 (9)	H16A—C16—H16C	109.5
O1—Mo1—N1	85.02 (8)	H16B—C16—H16C	109.5
N2—Mo1—N1	68.95 (8)	C15—C17—H17A	109.5
O3—Mo1—Cl1	92.21 (8)	C15—C17—H17B	109.5
O2—Mo1—Cl1	90.71 (7)	H17A—C17—H17B	109.5
O1—Mo1—Cl1	159.37 (6)	C15—C17—H17C	109.5
N2—Mo1—Cl1	76.36 (6)	H17A—C17—H17C	109.5
N1—Mo1—Cl1	77.70 (6)	H17B—C17—H17C	109.5
O5—Mo2—O4	107.35 (10)	C15—C18—H18A	109.5
O5—Mo2—O1	100.46 (10)	C15—C18—H18B	109.5
O4—Mo2—O1	99.73 (9)	H18A—C18—H18B	109.5
O5—Mo2—N4	159.56 (9)	C15—C18—H18C	109.5
O4—Mo2—N4	92.48 (9)	H18A—C18—H18C	109.5
O1—Mo2—N4	80.46 (8)	H18B—C18—H18C	109.5
O5—Mo2—N3	90.52 (9)	N3—C19—C20	122.7 (3)
O4—Mo2—N3	160.69 (9)	N3—C19—H19	118.7
O1—Mo2—N3	83.69 (8)	C20—C19—H19	118.7
N4—Mo2—N3	69.21 (8)	C19—C20—C21	120.7 (3)
O5—Mo2—Cl2	94.77 (8)	C19—C20—H20	119.7
O4—Mo2—Cl2	91.29 (7)	C21—C20—H20	119.7
O1—Mo2—Cl2	157.51 (6)	C20—C21—C22	116.2 (3)
N4—Mo2—Cl2	79.53 (6)	C20—C21—C29	123.0 (3)
N3—Mo2—Cl2	79.71 (6)	C22—C21—C29	120.8 (3)
C1—N1—C5	117.1 (2)	C21—C22—C23	121.1 (3)
C1—N1—Mo1	121.85 (18)	C21—C22—H22	119.4
C5—N1—Mo1	119.91 (18)	C23—C22—H22	119.4
C10—N2—C6	118.2 (2)	N3—C23—C22	121.3 (3)
C10—N2—Mo1	121.46 (19)	N3—C23—C24	115.0 (2)
C6—N2—Mo1	120.28 (18)	C22—C23—C24	123.6 (3)
C19—N3—C23	117.9 (2)	N4—C24—C25	121.7 (2)
C19—N3—Mo2	122.4 (2)	N4—C24—C23	115.1 (2)
C23—N3—Mo2	119.71 (17)	C25—C24—C23	123.2 (2)
C28—N4—C24	117.8 (2)	C24—C25—C26	120.9 (2)
C28—N4—Mo2	121.53 (18)	C24—C25—H25	119.6
C24—N4—Mo2	120.46 (18)	C26—C25—H25	119.6
Mo1—O1—Mo2	143.50 (10)	C27—C26—C25	116.2 (3)
N1—C1—C2	123.7 (3)	C27—C26—C33	123.6 (2)
N1—C1—H1	118.2	C25—C26—C33	120.1 (2)
C2—C1—H1	118.2	C28—C27—C26	120.1 (3)
C1—C2—C3	119.9 (3)	C28—C27—H27	119.9
C1—C2—H2	120.0	C26—C27—H27	119.9
C3—C2—H2	120.0	N4—C28—C27	123.3 (3)
C2—C3—C4	116.0 (3)	N4—C28—H28	118.4
C2—C3—C11	124.3 (3)	C27—C28—H28	118.4
C4—C3—C11	119.6 (3)	C31—C29—C32	109.0 (3)
C5—C4—C3	121.2 (3)	C31—C29—C30	109.2 (3)
C5—C4—H4	119.4	C32—C29—C30	109.2 (3)
C3—C4—H4	119.4	C31—C29—C21	111.2 (3)
N1—C5—C4	121.8 (3)	C32—C29—C21	110.2 (3)
N1—C5—C6	114.9 (2)	C30—C29—C21	108.1 (2)
C4—C5—C6	123.0 (3)	C29—C30—H30A	109.5
N2—C6—C7	121.4 (3)	C29—C30—H30B	109.5
N2—C6—C5	115.4 (2)	H30A—C30—H30B	109.5
C7—C6—C5	123.1 (3)	C29—C30—H30C	109.5
C6—C7—C8	120.3 (3)	H30A—C30—H30C	109.5
C6—C7—H7	119.8	H30B—C30—H30C	109.5
C8—C7—H7	119.8	C29—C31—H31A	109.5
C9—C8—C7	117.0 (3)	C29—C31—H31B	109.5
C9—C8—C15	123.1 (3)	H31A—C31—H31B	109.5
C7—C8—C15	119.9 (3)	C29—C31—H31C	109.5
C8—C9—C10	120.0 (3)	H31A—C31—H31C	109.5
C8—C9—H9	120.0	H31B—C31—H31C	109.5
C10—C9—H9	120.0	C29—C32—H32A	109.5
N2—C10—C9	123.0 (3)	C29—C32—H32B	109.5
N2—C10—H10	118.5	H32A—C32—H32B	109.5
C9—C10—H10	118.5	C29—C32—H32C	109.5
C14—C11—C3	111.8 (3)	H32A—C32—H32C	109.5
C14—C11—C13	109.9 (3)	H32B—C32—H32C	109.5
C3—C11—C13	108.6 (3)	C34—C33—C26	108.7 (3)
C14—C11—C12	108.4 (3)	C34—C33—C35	108.2 (3)
C3—C11—C12	108.5 (3)	C26—C33—C35	112.2 (2)
C13—C11—C12	109.8 (3)	C34—C33—C36	110.4 (3)
C11—C12—H12A	109.5	C26—C33—C36	108.5 (3)
C11—C12—H12B	109.5	C35—C33—C36	108.8 (3)
H12A—C12—H12B	109.5	C33—C34—H34A	109.5
C11—C12—H12C	109.5	C33—C34—H34B	109.5
H12A—C12—H12C	109.5	H34A—C34—H34B	109.5
H12B—C12—H12C	109.5	C33—C34—H34C	109.5
C11—C13—H13A	109.5	H34A—C34—H34C	109.5
C11—C13—H13B	109.5	H34B—C34—H34C	109.5
H13A—C13—H13B	109.5	C33—C35—H35A	109.5
C11—C13—H13C	109.5	C33—C35—H35B	109.5
H13A—C13—H13C	109.5	H35A—C35—H35B	109.5
H13B—C13—H13C	109.5	C33—C35—H35C	109.5
C11—C14—H14A	109.5	H35A—C35—H35C	109.5
C11—C14—H14B	109.5	H35B—C35—H35C	109.5
H14A—C14—H14B	109.5	C33—C36—H36A	109.5
C11—C14—H14C	109.5	C33—C36—H36B	109.5
H14A—C14—H14C	109.5	H36A—C36—H36B	109.5
H14B—C14—H14C	109.5	C33—C36—H36C	109.5
C18—C15—C16	114.4 (4)	H36A—C36—H36C	109.5
C18—C15—C8	110.3 (3)	H36B—C36—H36C	109.5
C16—C15—C8	110.9 (3)		
			
O3—Mo1—N1—C1	−154.4 (3)	N1—C5—C6—N2	−8.0 (4)
O2—Mo1—N1—C1	−2.2 (2)	C4—C5—C6—N2	166.9 (3)
O1—Mo1—N1—C1	98.8 (2)	N1—C5—C6—C7	174.8 (3)
N2—Mo1—N1—C1	−172.5 (2)	C4—C5—C6—C7	−10.3 (5)
Cl1—Mo1—N1—C1	−92.6 (2)	N2—C6—C7—C8	−3.5 (5)
O3—Mo1—N1—C5	13.1 (4)	C5—C6—C7—C8	173.5 (3)
O2—Mo1—N1—C5	165.3 (2)	C6—C7—C8—C9	1.2 (5)
O1—Mo1—N1—C5	−93.7 (2)	C6—C7—C8—C15	−179.8 (3)
N2—Mo1—N1—C5	−5.0 (2)	C7—C8—C9—C10	0.8 (5)
Cl1—Mo1—N1—C5	74.9 (2)	C15—C8—C9—C10	−178.2 (3)
O3—Mo1—N2—C10	3.8 (2)	C6—N2—C10—C9	−1.7 (4)
O2—Mo1—N2—C10	150.5 (3)	Mo1—N2—C10—C9	−179.0 (2)
O1—Mo1—N2—C10	−96.7 (2)	C8—C9—C10—N2	−0.6 (5)
N1—Mo1—N2—C10	177.5 (2)	C2—C3—C11—C14	5.6 (5)
Cl1—Mo1—N2—C10	95.7 (2)	C4—C3—C11—C14	−171.7 (3)
O3—Mo1—N2—C6	−173.5 (2)	C2—C3—C11—C13	−115.8 (4)
O2—Mo1—N2—C6	−26.7 (4)	C4—C3—C11—C13	67.0 (4)
O1—Mo1—N2—C6	86.1 (2)	C2—C3—C11—C12	125.0 (4)
N1—Mo1—N2—C6	0.3 (2)	C4—C3—C11—C12	−52.2 (4)
Cl1—Mo1—N2—C6	−81.6 (2)	C9—C8—C15—C18	−138.6 (4)
O5—Mo2—N3—C19	−1.0 (2)	C7—C8—C15—C18	42.5 (5)
O4—Mo2—N3—C19	157.1 (3)	C9—C8—C15—C16	−10.8 (5)
O1—Mo2—N3—C19	−101.5 (2)	C7—C8—C15—C16	170.3 (4)
N4—Mo2—N3—C19	176.4 (2)	C9—C8—C15—C17	106.4 (4)
Cl2—Mo2—N3—C19	93.7 (2)	C7—C8—C15—C17	−72.6 (4)
O5—Mo2—N3—C23	179.7 (2)	C23—N3—C19—C20	2.2 (4)
O4—Mo2—N3—C23	−22.2 (4)	Mo2—N3—C19—C20	−177.1 (2)
O1—Mo2—N3—C23	79.2 (2)	N3—C19—C20—C21	0.1 (5)
N4—Mo2—N3—C23	−3.0 (2)	C19—C20—C21—C22	−2.8 (4)
Cl2—Mo2—N3—C23	−85.6 (2)	C19—C20—C21—C29	177.6 (3)
O5—Mo2—N4—C28	−171.6 (3)	C20—C21—C22—C23	3.2 (4)
O4—Mo2—N4—C28	−5.4 (2)	C29—C21—C22—C23	−177.2 (3)
O1—Mo2—N4—C28	94.0 (2)	C19—N3—C23—C22	−1.8 (4)
N3—Mo2—N4—C28	−179.2 (2)	Mo2—N3—C23—C22	177.6 (2)
Cl2—Mo2—N4—C28	−96.3 (2)	C19—N3—C23—C24	−179.5 (3)
O5—Mo2—N4—C24	13.8 (4)	Mo2—N3—C23—C24	−0.1 (3)
O4—Mo2—N4—C24	179.9 (2)	C21—C22—C23—N3	−1.0 (4)
O1—Mo2—N4—C24	−80.6 (2)	C21—C22—C23—C24	176.5 (3)
N3—Mo2—N4—C24	6.2 (2)	C28—N4—C24—C25	−2.3 (4)
Cl2—Mo2—N4—C24	89.1 (2)	Mo2—N4—C24—C25	172.5 (2)
O3—Mo1—O1—Mo2	60.75 (19)	C28—N4—C24—C23	176.7 (2)
O2—Mo1—O1—Mo2	−48.57 (19)	Mo2—N4—C24—C23	−8.5 (3)
N2—Mo1—O1—Mo2	151.74 (18)	N3—C23—C24—N4	5.4 (4)
N1—Mo1—O1—Mo2	−139.15 (18)	C22—C23—C24—N4	−172.2 (3)
Cl1—Mo1—O1—Mo2	−172.16 (6)	N3—C23—C24—C25	−175.6 (3)
O5—Mo2—O1—Mo1	−40.34 (19)	C22—C23—C24—C25	6.8 (4)
O4—Mo2—O1—Mo1	−150.18 (18)	N4—C24—C25—C26	2.1 (4)
N4—Mo2—O1—Mo1	118.93 (18)	C23—C24—C25—C26	−176.8 (3)
N3—Mo2—O1—Mo1	49.02 (18)	C24—C25—C26—C27	−0.1 (4)
Cl2—Mo2—O1—Mo1	91.5 (2)	C24—C25—C26—C33	179.6 (3)
C5—N1—C1—C2	−3.3 (4)	C25—C26—C27—C28	−1.7 (4)
Mo1—N1—C1—C2	164.5 (2)	C33—C26—C27—C28	178.7 (3)
N1—C1—C2—C3	0.4 (5)	C24—N4—C28—C27	0.5 (4)
C1—C2—C3—C4	3.8 (4)	Mo2—N4—C28—C27	−174.3 (2)
C1—C2—C3—C11	−173.6 (3)	C26—C27—C28—N4	1.5 (5)
C2—C3—C4—C5	−5.2 (5)	C20—C21—C29—C31	−18.8 (4)
C11—C3—C4—C5	172.3 (3)	C22—C21—C29—C31	161.6 (3)
C1—N1—C5—C4	1.8 (4)	C20—C21—C29—C32	−139.8 (3)
Mo1—N1—C5—C4	−166.3 (2)	C22—C21—C29—C32	40.6 (4)
C1—N1—C5—C6	176.7 (2)	C20—C21—C29—C30	101.0 (4)
Mo1—N1—C5—C6	8.6 (3)	C22—C21—C29—C30	−78.6 (4)
C3—C4—C5—N1	2.6 (5)	C27—C26—C33—C34	−117.4 (3)
C3—C4—C5—C6	−172.0 (3)	C25—C26—C33—C34	62.9 (3)
C10—N2—C6—C7	3.7 (4)	C27—C26—C33—C35	2.2 (4)
Mo1—N2—C6—C7	−178.9 (2)	C25—C26—C33—C35	−177.4 (3)
C10—N2—C6—C5	−173.5 (2)	C27—C26—C33—C36	122.4 (3)
Mo1—N2—C6—C5	3.8 (3)	C25—C26—C33—C36	−57.2 (4)

### Hydrogen-bond geometry (Å, °)

**Table:**

*D*—H···*A*	*D*—H	H···*A*	*D*···*A*	*D*—H···*A*
C27—H27···O1^i^	0.95	2.52	3.341 (3)	145
C34—H34A···Cl1^ii^	0.98	2.77	3.748 (4)	174
C35—H35A···O4^i^	0.98	2.54	3.421 (4)	149
C12—H12C···O1W	0.98	2.69	3.641 (16)	163
C18—H18B···O1W	0.98	2.10	2.970 (18)	147
O1W—···.Cl2^i^	.	.	3.573 (18)	.
O1W—···.O5^iii^	.	.	3.236 (17)	.

Symmetry codes: (i) −*x*, −*y*+1, −*z*; (ii) −*x*+1/2, *y*−1/2, −*z*+1/2; (iii) *x*−1/2, −*y*+3/2, *z*+1/2.
